# The Correlation Between NTCP rs2296651 Variant and Risk of Hepatitis B, Cirrhosis, and Hepatocellular Carcinoma in Iranian Population

**DOI:** 10.34172/mejdd.2025.433

**Published:** 2025-07-30

**Authors:** Hassan Akrami, Mohammad Reza Fattahi, Mastaneh Zeraatiannejad, Jamal Sarvari, Yousef Nikmanesh, Seyedeh Azra Shamsdin, Zahra Mansourabadi, Saeid Amirizadeh Fard, Nazanin Gohari

**Affiliations:** ^1^Gastroenterohepatology Research Center, Shiraz University of Medical Sciences, Shiraz, Iran; ^2^Department of Bacteriology and Virology, School of Medicine, Shiraz University of Medical Sciences, Shiraz, Iran; ^3^Department of Biology, College of Sciences, Shiraz University, Shiraz 71467-13565, Iran

**Keywords:** Hepatitis B, Cirrhosis, Hepatocellular carcinoma, Sodium taurocholate co-transporting polypeptide (NTCP), rs2296651

## Abstract

**Background:**

Millions of people are suffering from different types of liver diseases worldwide. Hepatitis B, cirrhosis, and hepatocellular carcinoma (HCC) are the leading causes of death caused by the hepatitis B virus (HBV). HBV needs to interact with the sodium taurocholate co-transporting polypeptide (NTCP) to enter the cytoplasm of the hepatocyte. single nucleotide polymorphism (SNP) with rs2296651 (Ser267Phe, S267F, G>A) variation in the NTCP has been investigated as a reverse association with the function of NTCP and entry of HBV into the cell in the Asian population. We investigated the relationship between the NTCP rs2296651 variant and HBV infection, liver cirrhosis, and HCC in the Iranian population.

**Methods:**

Whole blood DNA of 50 healthy individuals as a control group and 90 patients (HBV, cirrhosis, and HCC) were extracted, and the tetra amplification refractory mutation system polymerase chain reaction (tetra-ARMS PCR) was done to identify the genotypes of the samples.

**Results:**

Based on our analytical tests using SPSS software, there was a positive and significant association between NTCP rs2296651 in the control group and cirrhosis (*P*=0.002), as well as between cirrhosis and HBV (*P*<0.001). However, there was a negative relationship between cirrhosis and HCC (*P*=0.003).

**Conclusion:**

The NTCP rs2296651 variant may confer resistance to HBV infection in the Iranian population.

## Introduction

 Hepatitis B is a liver disease caused by the hepatitis B virus (HBV) and exists as both an acute and chronic disease. The statistical data from 187 countries in 2022 revealed about 254 million people infected with hepatitis B, with higher prevalence in Asia, 1.2 million new cases, and 1.3 million deaths from hepatitis B.^1^ Chronic hepatitis B affects about 300 million people and has a high risk of death due to cirrhosis and hepatocellular carcinoma (HCC).^2^ The infection can be distributed by physical contact with infected body fluids or passed from a mother to child at birth.^3^

 HBV, a member of the Hepadnaviridae family and *Orthohepadnavirus* genus, has ~3.2 kb circular and partially double-stranded DNA that replicates by reverse transcription mechanism and encodes envelope proteins, core protein, polymerase, RNase H, and the regulatory X-protein.^4^ The entry of HBV to the cytoplasm is mediated by the interaction of the preS1 protein of HBV and the sodium taurocholate co-transporting polypeptide (NTCP) receptor of hepatocytes.^5^ Many studies have investigated the relationship between NTCP polymorphisms and the risk of HBV infection and HCC progression. One of the most investigated variants in NCTP in the Asian population is the rs2296651 variant (Ser267Phe, S267F, G > A), which can disrupt the function of the NTCP receptor in vitro and has a minor allele frequency ranging from 3.1% to 9.2% in Asian populations.^6^ A 2018 study demonstrated an association between the rs2296651 variation in NTCP and a decreased risk of HBV infection in Taiwanese women.^7^ Lina Wu et al. indicated that heterozygous alleles in rs2296651 supported the Nucleus(t)ide analogs treatment.^8^ Yang et al found that the NTCP rs2296651 variant was associated with a reduction in chronic HBV infection and an increased risk of cirrhosis and HCC.^9^ These investigations proposed that the rs2296651 variant may be related to chronic HBV infection and also the risk of cirrhosis and HCC progression in the East Asian population. However, some investigations have found an inverse relationship between the rs2296651 variant and HBV infection, as well as the risk of cirrhosis and HCC progression, contrary to other studies.^6,10^

 In this study, we examined the NTCP rs2296651 variant in patients with HBV infection, liver cirrhosis, and HCC to find an association between rs2296651 variation and the risk for HBV infection and HCC progression in the Iranian Population.

## Materials and Methods

###  Study Design

 The association between HCC incidence and NTCP rs2296651 variant was investigated in a study comprising healthy persons and patients with hepatitis B infection, liver cirrhosis, and HCC. 140 participants aged between 45 and 70 years including 50 healthy persons as controls, 50 patients with HBV infection, 20 with liver cirrhosis and 20 with HCC were involved in this study from Shiraz hospitals in Iran.

###  Sample Preparation and DNA Isolation

 Two milliliters of blood were drawn from each participant into an EDTA tube for DNA extraction. Genomic (g) DNA was extracted from the EDTA blood sample using Bioneer’s AccuPrep® Genomic DNA Extraction Kit as described in the manual product and preserved below – 20 °C.

###  Primer Design

 The primers of tetra Amplification Refractory Mutation System Polymerase Chain Reaction (tetra-ARMS PCR) for detecting NTCP rs2296651 variant (G > A) were designed using two web-based programs: Primer1 (http://primer1.soton.ac.uk/public_html/primer1.html) and tetra-ARMS PCR Primer Design Tool (https://snp.biotech.edu.lk/arms.php) (Table 1). The specificity and biophysical properties of primers were checked by BLAST (http://www.ncbi.nlm.nih.gov/blast) and Gene Runner (http://www.generunner.net) programs.

**Table 1:** Primer sequences for detection of NTCP rs2296651 variant (G > A) used in tetra-ARMS PCR

**Primers name**	**Sequences (5'→3')**	**Length (bp)**
FIA-2296651	5' - AAA GGC CAC ATT GAG GAT GGG GA-3'	23
RIG-2296651	5' -ATG CCA AAA TGT CCA ACT CTG TGC C-3'	25
Fout-2296651	5' -AGT CCC TGC TAG AAA CTT GCT TGT TGG C-3'	28
Rout-2296651	5' -AGC AAA GTA CCC CTG TCC AGG GTC TAG A-3'	28

###  Tetra-ARMS PCR and Genotyping

 Tetra-ARMS PCR was carried out using Taq DNA Polymerase Master Mix (2X) (Ampliqon A/S, Denmark) and 100 ng of DNA sample, 1 mM of both outer primers, 0.5 mM of both inner primers, 1 µL MgCl_2_ (25 mM) and 0.5 µL DMSO in a total volume of 10 μL. The thermocycler program was optimized as follows: an initial denaturation at 94 ˚C for 4 minutes, followed by 40 cycles of denaturation at 95 ˚C for 30 seconds, annealing at 65˚C for 20 seconds, and extension at 72 ˚C for 20 seconds and an additional extension at 72˚C for 5 min.

###  Gel Electrophoresis

 Tetra-ARMS PCR products were analyzed on 2.5% agarose gels by electrophoresis and stained with ethidium bromide dye. The PCR bands were visualized under UV light using a Gel Doc apparatus (Kimiagene Technology Company).

###  Statistical Analysis

 All statistical analyses were carried out using SPSS software, version 25 (SPSS Inc., Chicago, IL, USA). Diﬀerences in frequency of rs2296651 genotypes (GG, AG, AA) in different groups (control, HBV, cirrhosis, and HCC) and distribution deviation of the observed genotype in all groups from Hardy–Weinberg equilibrium were analyzed using the chi-square test. The correlation between combining GG and AG vs AA genotypes and the risk of different groups, along with a 95% confidence interval (CI), was surveyed using the odds ratio (OR). The genotype AA was considered as H^0^ and *P* value < 0.05 as a significant limit in the odds ratio (OR) test. The variable genotypes between groups were measured by one-way ANOVA, and the *P* value < 0.05. The heterogeneity of within-group variance was measured by Welch’s ANOVA test, and the *P* value < 0.05.

## Results

###  Demographic Information

 Overall, 140 samples were enrolled in this study, consisting of 50 healthy subjects with a mean age of 55.44 years, 50 patients with HBV with a mean age of 53.2 years, 20 patients with Cirrhosis with a mean age of 52.55 years, and 20 patients with HCC with a mean age of 55 years. The age and sex-related characteristics of the different groups are presented in Table 2. Statistically significant differences observed in the age and sex-related characteristics between different groups based on the calculated *P* value < 0.05.

**Table 2:** The distributions of NTCP rs2296651 variant polymorphism genotype in different groups

**Groups **	**Age (y)**	**Sex**		**Genotypes**			**Alleles**	
		**F**	**M**	**GG**	**AG**	**AA**	**G**	**A**
Control	55.44	23	27	25	18	7	68	32
HBV	53.2	26	24	28	16	6	73	27
Cirrhosis	52.55	8	12	4	5	11	13	27
HCC	55	9	11	12	4	4	29	11

###  Tetra-ARMS PCR 

**Figure 1:**
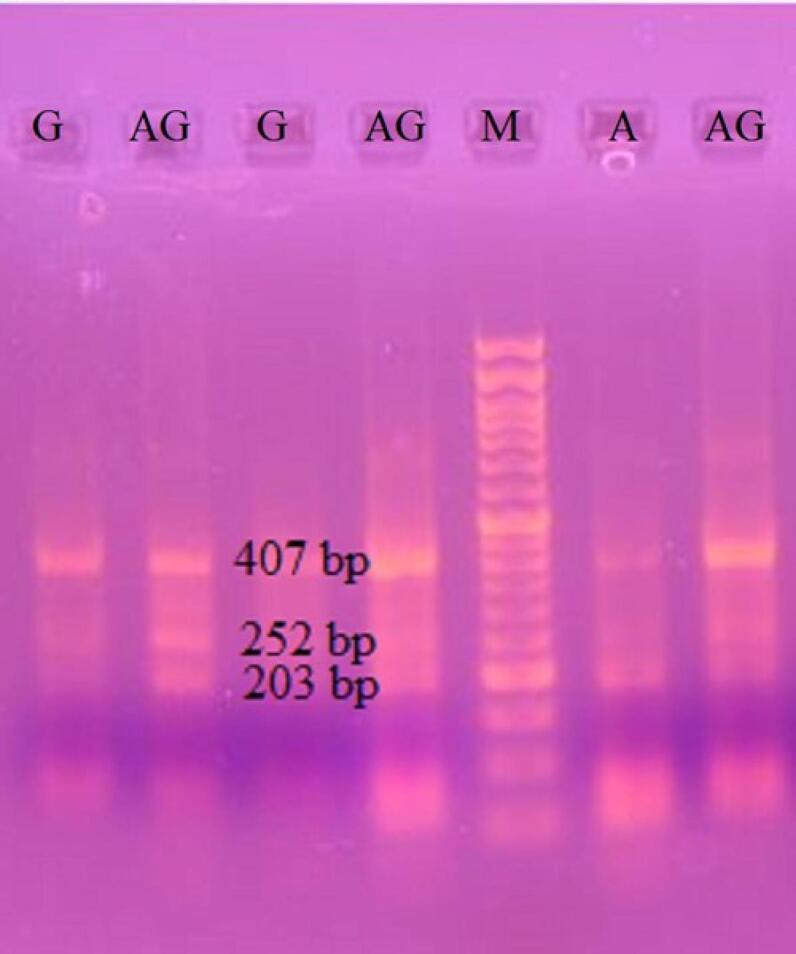
The tetra-ARMS PCR results of 8 samples with NTCP rs2296651 genotypes polymorphism. The common product size was 407 bp. The product size of G allele represented a 252 bp band, and A allele represented a 203 bp band on 2.5% gel electrophoresis. M: 50 bp DNA Ladder (SMOBIO Technology, Inc., Taiwan)

 The common product size of tetra-ARMS PCR (outer forward and outer reverse) was 407 bp. The product size of the G allele (inner forward and outer reverse) represented a 252 bp band, and the A allele (outer forward and inner reverse) represented a 203 bp band on 2.5% gel electrophoresis (Figure 1).

###  NTCP rs2296651 Variant (G > A) Genotype Distributions

 The distributions of NTCP rs2296651 variant polymorphism genotype in different groups are listed in Table 2. Genotype frequencies are in accordance with the Hardy-Weinberg principle in all groups. As seen in Table 2, genotypes of NTCP rs2296651 variant were distributed as 25 GG (50%), 18 AG (36%) and 7 AA (14%) in the control or healthy group, 28 GG (56%), 16 AG (32%) and 6 AA (12%) in HBV group, 4 GG (20%), 5 AG (25%) and 11 AA (55%) in the cirrhosis group and 12 GG (60%), 4 AG (20%) and 4 AA (20%) in the HCC group. The genotype frequency showed significant differences between the cirrhosis and control groups, as well as between the HBV and HCC groups (*P* < 0.05); however, the genotype frequency between the control and HBV and HCC groups did not show any significant differences (*P* > 0.05) (Table 3).

**Table 3:** The differences in distributions between groups

**Cross groups**		**Mean difference (I-J)**	**Standard error**	**Sig.**
Control	HBV	0.109	0.145	0.451
	Cirrhosis	-.710^*^	0.190	0.001
	HCC	0.228	0.202	0.261
HBV	Control	-0.109	0.145	0.451
	Cirrhosis	-.819^*^	0.191	0.001
	HCC	0.119	0.203	0.558
Cirrhosis	Control	.710^*^	0.190	0.001
	HBV	.819^*^	0.191	0.001
	HCC	.938^*^	0.237	0.001
HCC	Control	-0.228	0.202	0.261
	HBV	-0.119	0.203	0.558
	Cirrhosis	-.938^*^	0.237	0.001

* The mean difference is significant at the 0.05 level.

 The correlation of the genetic change effect on different groups was calculated using the *P *value and OR tests. The results of *P *value and OR tests for GG, AG and AA genotypes in different groups indicated a significant positive correlation between genotypes in cirrhosis group and control and HBV groups (OR = 3.247, and *P* = 0.002 for control and cirrhosis, OR = 3.971, and *P* < 0.001 for HBV) and a significant negative correlation between genotypes in cirrhosis group and HCC group (cirrhosis, OR = 0.242, and *P* = 0.003 for cirrhosis and HCC); but we did not find any correlation between genotype in control, HBV and HCC groups (*P* > 0.05).

## Discussion

 Previous studies, especially in vitro and animal tests, have identified NTCP as the receptor of HBV on the surface of hepatocytes, and there are some functional SNPs associated with it. Based on these findings, NTCP rs2296651 S267F appears to exist only in the Asian population, and there is a relationship between the NTCP rs2296651 (S267F) and HBV uptake-related disorders. Individuals carrying phenylalanine instead of serine are susceptible to mitigating the transportation function of NTCP.^11,12^ A study on the group of Chinese Han population indicated A allele may play a protective role against chronic HBV infection.^13^ Additionally, the GA genotype in the Chinese population was associated with a decreased risk of metastasis in HCC.^14^ Based on a study of individuals, the NTCP rs2296651 AA + GA variations may decrease the risk of HBV infection and HCC progression.^15^ Thus, in this study, we evaluated whether this SNP has any significant correlation with HBV infection, cirrhosis, and HCC in the Iranian population. Tetra-ARMS PCR and SPSS data analysis revealed that rs2296651 AA homozygote genotype had a reverse correlation with cirrhosis and control (*P* = 0.002), cirrhosis and HCC (P = 0.003), and cirrhosis and HBV (*P* < 0.001). Same as previous results, our data are consistent with the fact that this SNP has a protective role against some HBV-related disorders. For instance, a meta-analysis conducted in China in 2017 revealed the same relationship between this polymorphism of NTCP and HBV infection.^16^ All in all, our data confirm it in the Iranian population that individuals with rs2296651 AA homozygote genotype may have resistance to HBV-related disorders. This can be explained by an in vitro study conducted in 2018, which discovered that AA homozygotes attenuate the HBV uptake ability of hepatocytes.^17^

 Cirrhosis is a common chronic liver disorder that causes parenchymal lesions; one of the important risk factors for this is HBV infection, and cirrhosis patients with HBV are more susceptible to suffering from HCC.^18^ And based on our data, cirrhotic patients with rs2296651 AA have a significant relationship with a lower development of HCC. The exact function of this variant has not been clear yet. However, it seems it is the consequence of alleviation of hepatic inflammation and oxidative stress-mediated tumorigenesis as a result of substituting Phenylalanine instead of serine in the NTCP receptor and diminishing cytotoxic bile salt accumulation in hepatocytes by deduction of bile acid uptake.^19,20^

 In conclusion, although our study had some limitations, the data confirmed the hypothesis that the NTCP S267F SNP plays a protective role in cirrhosis progression in the Iranian population. Due to the strong conclusions drawn about the impact of the S267F variant on the function of NTCP as a drug target, further studies are needed in West Asian populations, as well as clinical analysis, to determine the exact role behind its effect.
